# Unraveling a Rare Diagnosis: Genetically Confirmed Cornelia De Lange Syndrome in an Emirati Female Patient

**DOI:** 10.7759/cureus.115475

**Published:** 2026-08-31

**Authors:** Hany T Tobia, Fakhriya Alalawi, Dhanya Mohan, Amna Alhadari

**Affiliations:** 1 Department of Nephrology, Dubai Hospital, Dubai Academic Health Corporation, Dubai, ARE; 2 Department of Nephrology, Dubai Academic Health Corporation, Dubai, ARE; 3 Department of Nephrology, Dubai Health Authority, Dubai, ARE

**Keywords:** chronic kidney disease (ckd), cornelia de lange syndrome (cdls), developmental delay, epilepsy, nipbl gene

## Abstract

Cornelia de Lange syndrome (CdLS) is a rare multisystem genetic disorder characterized by distinctive facial features, developmental delay, and a range of congenital anomalies. Despite increasing recognition globally, detailed reports from the Arabian Gulf region remain limited, particularly those incorporating long-term clinical follow-up and genetic confirmation. Here, we report the case of a 28-year-old Emirati female who was clinically diagnosed with CdLS at birth and subsequently developed intractable epilepsy, profound developmental delay, hypothyroidism, stage 3 chronic kidney disease, and significant behavioral difficulties. The patient is bedridden, noncommunicative, and fully dependent on family care. Genetic testing identified a novel 4-base-pair frameshift insertion pathogenic variant impacting the NIPBL gene, confirming the clinical diagnosis and underscoring the diagnostic complexity, multifaceted management challenges, and the critical need for coordinated multidisciplinary care in patients with CdLS.

## Introduction

Cornelia de Lange syndrome (CdLS) is a rare, multisystem genetic disorder. It was named after the Dutch pediatrician Cornelia de Lange, who first described it in 1933 [1]. The prevalence of CdLS is estimated to be one in 10,000 to one in 30,000 [2].

Reports of CdLS in the Arabian Gulf region are scarce. To date, the literature includes a single case report from Saudi Arabia [3], one affected family described in Kuwait [4], and one case reported from Oman [5]. Notably, in a comprehensive analysis of major congenital anomalies in the United Arab Emirates population conducted by Al-Gazali et al. in 1995 [6], CdLS was not reported, further underscoring the rarity of this condition in the region or possibly reflecting underreporting of such cases.

Patients with CdLS may display craniofacial dysmorphisms (such as synophrys, a long philtrum, highly arched and/or thick eyebrows, long eyelashes, a short nasal bridge with anteverted nares, small widely spaced teeth, and microcephaly), hirsutism, limb anomalies, and upper-limb reduction defects ranging from subtle phalangeal abnormalities to oligodactyly (missing digits), developmental delays, seizures, and cognitive and behavioral characteristics. Cognitive characteristics in CdLS include brain-related functions such as memory, reasoning, and information processing, and these features can differ significantly between individuals, ranging from subtle variations to pronounced challenges [7-11]. Other common systemic findings include gastroesophageal reflux disease (GERD), pyloric stenosis (4%), and intestinal malrotation (2%) [8,12]. Congenital cardiac diseases have been reported in 25%-30% of cases, including septal defects, pulmonic or peripheral pulmonic stenosis, coarctation or hypoplastic aortic arch, tetralogy of Fallot, double-outlet right ventricle, and atrioventricular canal defect [13]. Other reported features include chronic ear infections, hearing loss, myopia, cryptorchidism, and hypoplastic genitalia [7-10]. Several genetic mutations have been linked to the syndrome, with NIPBL mutations responsible for the majority of cases [11,14,15].

Here, we report a genetically confirmed case of CdLS with significant multisystem involvement, highlighting the complexities associated with long-term management.

## Case presentation

A 28-year-old Emirati female, diagnosed with CdLS since birth (Figure 1 shows characteristic CdLS craniofacial features), was on regular follow-up at the Neurology Clinic. Her medical history was significant for medically refractory generalized epilepsy and global developmental delay. She is bedridden, noncommunicative, and fully dependent on her family for activities of daily living, requiring 24/7 care. The patient had a history of self-injurious behavior, recurrent episodes of anger outbursts, and physical aggression. She also suffered from sleep disturbances, primarily insomnia. Her medical history also included thalassemia trait, hypothyroidism treated with thyroxine replacement and followed by an endocrinologist, severe dental caries, frequent episodes of GERD, and a hysterectomy performed for severe menorrhagia. Additionally, she had recurrent infections, including frequent chest and urinary tract infections. Her transthoracic echocardiogram in 2024 was reported to be normal, with overall normal left ventricular systolic function and an ejection fraction of 60%-65%. No septal defects or valvular lesions were documented, and there was no evidence of pulmonary hypertension.

**Figure 1:**
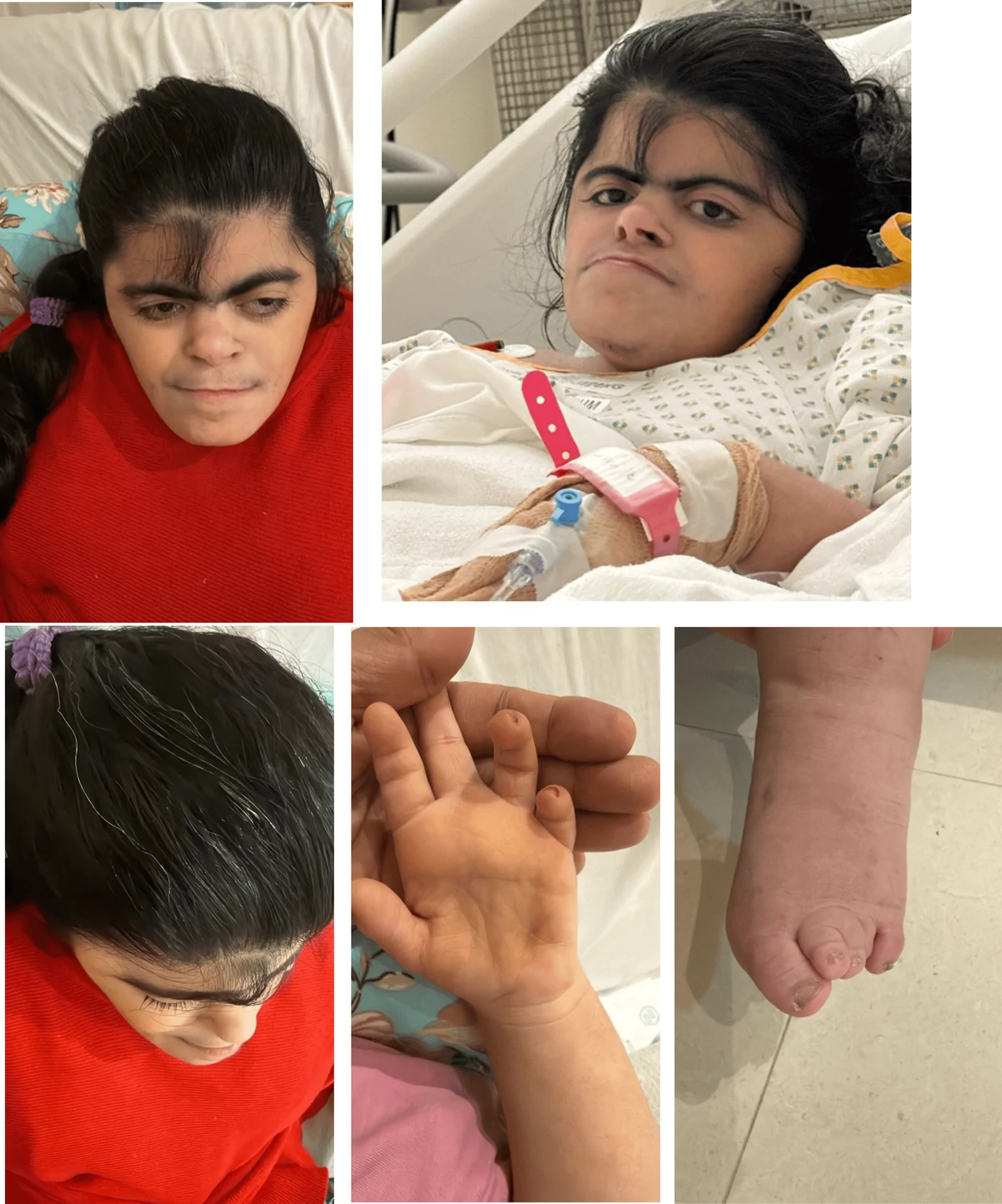
Our patient has characteristic facial features of CdLS, evident as thick, high, arched, and merging eyebrows (synophrys), thick, long eyelashes, an upturned nose with anteverted nares, a long, smooth philtrum, a thin upper lip with downturned corners of the mouth, and low-set ears. She also had low anterior and posterior hairlines, often accompanied by hirsutism, small hands and feet, short, tapering fingers with syndactyly, and premature graying of the hair (canities) CdLS: Cornelia de Lange syndrome Written informed consent for publication of this case report and any accompanying images was obtained from the patient’s legally authorized representative.

In September 2024, she was referred to the Nephrology Clinic due to renal impairment. Laboratory investigations revealed a creatinine level of 1.66 mg/dL and an estimated glomerular filtration rate (eGFR) of 43.6 mL/min/1.73 m², consistent with stage 3b chronic kidney disease (CKD). Table 1 includes the laboratory parameters upon presentation. Ultrasound for kidneys, ureters, and bladder (Figure 2) performed in October 2024 revealed relatively small-sized, echogenic kidneys bilaterally. The right kidney measured 7.7 cm × 4.2 cm, with a parenchymal thickness of 1.8 cm, while the left kidney measured 7.8 cm × 4.2 cm, with a parenchymal thickness of 1.5 cm. Corticomedullary differentiation was maintained. There were no obvious calculi or hydronephrosis. A small exophytic cortical cyst was seen arising from the lower pole of the left kidney, measuring 12 mm × 10 mm, with a thin internal septum (Bosniak II). The urinary bladder was empty at the time of the scan. The patient was further evaluated at the Dubai Nephrology Genetic Clinic, where congenital anomalies of the kidney and urinary tract (CAKUT) genetic testing, including a 251-gene panel, identified a heterozygous likely pathogenic variant, classified according to The American College of Medical Genetics and Genomics and the Association for Molecular Pathology guidelines, c.1036_1040dup (p.Ile347MetfsTer3), in the NIPBL gene, with autosomal dominant inheritance, confirming the diagnosis of CdLS. It is important to note that there was no family history of CdLS among the patient's parents or siblings, and her parents were nonconsanguineous.

**Table 1:** Patients' labs FT4: free thyroxine; TSH: thyroid-stimulating hormone; FBC: full blood count; CBC: complete blood count; DIFF: differential test; MCV: mean corpuscular volume; MCH: mean corpuscular hemoglobin; MCHC: mean corpuscular hemoglobin concentration; RDW: red cell distribution width; eGFR: estimated glomerular filtration rate; CKD-EPI: chronic kidney disease epidemiology collaboration equation; SGPT: serum glutamic-pyruvic transaminase; ALT: alanine aminotransferase

Thyroid function test (FT4, TSH)			
	Collection time: 22/08/24 12:00		
Result		Value	Ref. range
	Free T4	11.2 (L)	(12.0-22.0) pmol/L
	TSH	28.230 (H)	(0.3-4.2) µIU/mL
FBC (CBC+DIFF)			
	Collection time: 22/08/24 12:00		
Result		Value	Ref. range
	WBC count	5.9	(3.6-11.0)×10^3^/uL
	RBC count	4.68	(3.80-4.80)×10^6^/uL
	Hemoglobin, blood	9.9 (L)	(12.0-15.0) g/dL
	Hematocrit	31.0 (L)	36.0%-46.0%
	MCV	66.2 (L)	(77.0-95.0) fL
	MCH	21.2 (L)	(27.0-32.0) pg
	MCHC	32.0	(31.5-34.5) g/dL
	RDW	16.3 (H)	11.5%-14.0%
	Platelet count	185	(150-410)×10^3^/uL
Creatinine blood			
	Collection time: 22/08/24 12:00		
Result		Value	Ref. range
	Creatinine	1.79 (H)	(0.50-0.90) mg/dL
	eGFR (CKD-EPI)	39.6 (L)	>60 mL/min/1.73m(2)
Liver function test			
	Collection time: 22/08/24 12:00		
Result		Value	Ref. range
	Bilirubin, Total	0.17	(0-1.0) mg/dL
	Alkaline phosphatase	68	(35-104) U/L
	SGPT(ALT)	11	(0-31) U/L
	Total Protein	7.3	(6.6-8.7) g/dL
	Albumin	3.5	(3.4-4.8) g/dL
	Globulin	3.8 (H)	(2.8-3.4) g/dL
Urea electrolytes			
	Collection time: 22/08/24 12:00		
Result		Value	Ref. range
	Sodium	130 (L)	(136-145) mmol/L
	Potassium	4.8	(3.3-4.8) mmol/L
	Chloride	99	(98 -108) mmol/L
	Bicarbonate (HCO_3_)	21.6	(20-28) mmol/L
	Urea	37	(12-40) mg/dL
	Anion gap	9	(6-14) mmol/L

**Figure 2:**
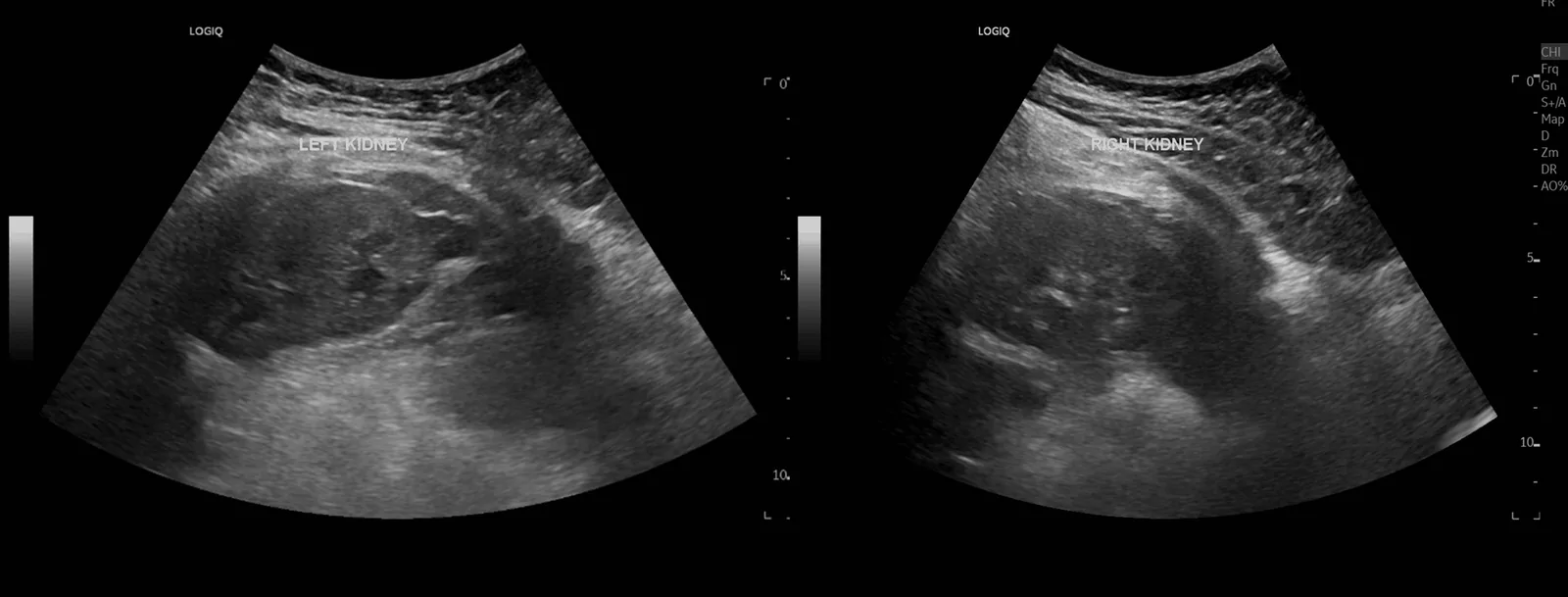
Both kidneys are relatively small in size and echogenic, with maintained corticomedullary differentiation

## Discussion

CdLS is a complex disorder that presents considerable management challenges during the transition to adulthood, as in our patient, who exhibited typical signs of global developmental delay, severe behavioral issues, and various systemic problems [16]. The presence of a pathogenic NIPBL variant confirms the clinical diagnosis, since NIPBL mutations are the most prevalent genetic cause of CdLS.

Urinary tract anomalies are common in CdLS, affecting approximately 12% to 41% of patients. Kidney abnormalities primarily include vesicoureteral reflux, small kidneys (hypoplasia), renal ectopia, and isolated renal cysts (as in our patient). While 85% of individuals maintain normal renal function, about 15% may experience impaired renal function or proteinuria, and 1% of cases may progress to renal failure [17,18]. Cholelithiasis and nephrolithiasis have also been reported [19]. The presence of CKD in this patient is likely multifactorial, related primarily to recurrent urinary tract infections, possibly leading to chronic pyelonephritis, long-term medication exposure, and limited mobility.

Behavioral disturbances such as autistic features, self-injury, and aggression are well-recognized features of CdLS and contribute substantially to the complexity of care and the psychological burden on caregivers [7].

A diagnosis of CdLS necessitates lifelong medical, multidisciplinary, and social care. The involvement of individuals with CdLS and their caregivers in healthcare decisions is essential [16]. Access to clinical evaluation, counseling, and follow-up by a multidisciplinary team is likely to improve healthcare and quality of life. Individuals with CdLS are now likely to live into adulthood, owing to improved care, and even into old age, and thus are at risk of developing common chronic diseases in addition to CdLS-related morbidities, such as heart or gastrointestinal issues [20]. Life expectancy is largely dependent on the severity of associated medical conditions, while mortality can occur, commonly as a result of aspiration/reflux and pneumonia in 31% of cases, followed by gastrointestinal disease, including obstruction/volvulus (19%). Congenital anomalies accounted for 15% of deaths and included congenital diaphragmatic hernia and congenital heart defects [18].

The uniqueness of this case extends beyond the rarity of CdLS in the region. To the best of our knowledge, this is the first reported case of genetically confirmed CdLS in the United Arab Emirates, with long-term clinical follow-up demonstrating the multisystem nature of the disorder. Importantly, most of the published literature on CdLS focuses on children, whereas detailed descriptions of adult patients and their long-term clinical course remain limited.

## Conclusions

This case underscores the intricate clinical challenges faced by adults with CdLS, highlighting the importance of a multidisciplinary approach to managing related health issues, including neurology, nephrology, endocrinology, and social support, to effectively meet the diverse needs of patients with CdLS throughout their lives. Genetic confirmation is vital for accurate diagnosis, prognosis, and family counseling.
